# Identification of potential active ingredients from *Chuanxiong Rhizoma* in the treatment of vascular dementia: CHI3L1 and TLR2 are potential key targets

**DOI:** 10.1097/MD.0000000000051414

**Published:** 2026-11-06

**Authors:** Hongchun Liao, Qimin Zeng, Jie Zhang, Taolin Chen, Juan Li, Qi He, Linjie Sun, Jing Wang, Binsheng He, Qin Xiang

**Affiliations:** a Hunan Provincial University Key Laboratory of the Fundamental and Clinical Research on Neurodegenerative Diseases, Changsha Medical University, Changsha, Hunan, China; b Hunan Provincial Key Laboratory of the Traditional Chinese Medicine Agricultural Biogenomics, Changsha Medical University, Changsha, Hunan, China; c College of Basic Medicine, Changsha Medical University, Changsha, Hunan, China; d Department of Clinical Psychology, Ziyang District Brain Hospital, Yiyang, Hunan, China; e Department of Radiology, Lixian People’s Hospital, Changde, Hunan, China.

**Keywords:** CHI3L1, *Chuanxiong Rhizoma*, integrated bioinformatics, TLR2, vascular dementia

## Abstract

Vascular dementia (VaD) is a cognitive impairment resulting from cerebrovascular disease, which causes functional decline. Its rising prevalence and the absence of effective therapeutic interventions highlight an urgent need for novel therapeutic strategies. The research aimed to identify crucial target genes and bioactive compounds responsible for the therapeutic effects of *Chuanxiong Rhizoma* in VaD through a comprehensive bioinformatics strategy. Network pharmacology identified 240 shared target genes between *Chuanxiong Rhizoma* and VaD. Weighted gene co-expression network analysis identified 114 genes significantly associated with VaD. Venn analysis identified CHI3L1, TAC1, toll-like receptor 2 (TLR2), and CCL2 as candidate target genes. Differential expression analysis confirmed dysregulation of CHI3L1, TAC1, TLR2, and CCL2 in VaD. Single-cell RNA analysis showed CHI3L1 predominantly in fibrous astrocytes, TAC1 in Parvalbumin inhibitory neurons, and TLR2 in microglia. Receiver operating characteristic analysis showed CHI3L1 (area under the curve [AUC] = 0.6597, *P* = .0144), TAC1 (AUC = 0.7797, *P* < .0001), and TLR2 (AUC = 0.7468, *P* = .0002) had significant AUC values, indicating potential diagnostic value for VaD. Gene set enrichment analysis showed CHI3L1, TAC1, and TLR2 enriched in chemokine signaling, allograft rejection, and JAK/STAT pathways. Immune cell infiltration analysis indicated that the correlation of CHI3L1 with Macrophages M2 was significantly positive, whereas it was significantly negative with activated natural killer cells (NK cells). The correlation of TAC1 with Macrophages M2 was significantly negative, whereas it was significantly positive with both activated NK cells and follicular helper T cells. The correlation of TLR2 with Macrophages M2 was significantly positive, whereas it was significantly negative with activated NK cells and follicular helper T cells. Molecular docking followed by 100-nanosecond molecular dynamics simulations indicated that palmitic acid binds stably to both CHI3L1 and TLR2, and that perillyl alcohol binds stably to TLR2, whereas the CHI3L1-perillyl alcohol complex showed persistent instability during the molecular dynamics trajectory and was therefore not considered a supported interaction. The findings indicated *Chuanxiong Rhizoma* might exert its anti-VaD effects through palmitic acid and perillyl alcohol by targeting CHI3L1 and TLR2. The study generated a multi-component, multi-target hypothesis for VaD that warrants further experimental validation.

## 1. Introduction

Vascular dementia (VaD) is a cognitive disorder related to cerebrovascular conditions, characterized by clinical impairment of cognitive function, and ranks as the second most prevalent form of dementia following Alzheimer’s disease (AD).^[1,2]^ VaD results from occlusion or narrowing of cerebral blood vessels, leading to reduced cerebral perfusion and subsequent brain tissue injury.^[3,4]^ In Asia and other developing regions, it accounts for roughly 30 percent of all dementia cases.^[5]^ Hypertension, diabetes, and hyperlipidemia are major contributors to the risk of VaD.^[6]^ Unlike AD, VaD typically presents with an abrupt onset and a stepwise progression pattern. In addition to cognitive deficits like memory loss, patients frequently exhibit neurological symptoms, including difficulties with speech, paralysis of the face, hemiplegia, balance dysfunction, and abnormal gait.^[7,8]^ Currently, there are few approved pharmacological treatments for VaD, largely due to its heterogeneous clinical manifestations and complex pathophysiology.^[7]^ Consequently, there is a critical necessity for research focused on the development of innovative therapeutic agents for VaD. Such agents are expected to act on multiple targets simultaneously, and future drug development should focus on modulating key pathological processes such as inflammation, apoptosis, and cell adhesion.

Herbal remedies are extensively employed in the treatment of VaD in Asian nations, notably in China and Korea.^[6]^ *Chuanxiong Rhizoma,* obtained from the parched rhizome of *Ligusticum chuanxiong Hort.*, is an essential element in traditional Chinese medicine (TCM), traditionally employed to improve circulation and reduce blood stagnation.^[9]^ Its medicinal use is documented as early as the Shennong’s Herbal Classic, where it is described as having pungent and warm properties.^[10]^ This herb exhibits broad clinical efficacy in treating disorders of the cardiovascular, cerebrovascular, nervous, respiratory, digestive, and urinary systems, attributed to its anti-ischemic, anti-inflammatory properties, its antioxidant capabilities, its neuroprotective benefits, and its antitumor actions.^[11–13]^ In TCM practice, *Chuanxiong Rhizoma* is commonly used to invigorate blood, regulate qi, dispel wind, and relieve pain.^[9]^ To date, over 200 chemical constituents have been isolated from *Chuanxiong Rhizoma*, encompassing phthalides, organic acids, alkaloids, polysaccharides, and various other biologically active compounds.^[9]^ Notably, several small-molecule components, like ligustilide, ferulic acid, as well as senkyunolides I and A, have indicated the ability to penetrate the blood-brain barrier and provide substantial neuroprotective benefits.^[14,15]^ Additionally, *Chuanxiong Rhizoma* possesses anti-neuroinflammatory properties, with individual compounds like ligustilide, senkyunolide I, and senkyunolide H shown to protect neuronal cells against damage.^[16,17]^ Extracts of *Chuanxiong Rhizoma* also show promising therapeutic potential in Parkinson’s disease.^[18]^ Although evidence suggests its effectiveness in conditions such as cerebral infarction and VaD, the underlying molecular mechanisms remain poorly elucidated. Therefore, identifying the key therapeutic targets and active ingredients of *Chuanxiong Rhizoma* in the context of VaD treatment holds significant clinical and pharmacological relevance.

Our study sought to identify the molecular targets and potential bioactive constituents of *Chuanxiong Rhizoma* in the treatment of VaD using an integrated bioinformatics approach. The strategy integrated weighted gene co-expression network analysis (WGCNA), differential expression analysis, single-cell expression profiling, receiver operating characteristic (ROC) analysis, gene set enrichment analysis (GSEA), immune infiltration assessment, molecular docking, and molecular dynamics simulations. Although WGCNA of the human brain dataset analyzed here (GSE122063) has previously implicated toll-like receptor 2 (TLR2) in VaD-related neuroinflammation, the present study extends that work by intersecting the disease-associated module with the herb’s chemical constituents, resolving candidate genes to individual brain cell types, and testing each compound-target pair by molecular dynamics simulation.^[19]^ Our results therefore nominate chitinase 3-like protein 1 (CHI3L1) and TLR2 as potential key therapeutic targets, with perillyl alcohol and palmitic acid as candidate active compounds that may contribute to the pharmacological mechanisms of *Chuanxiong Rhizoma* in VaD. These findings generate testable hypotheses regarding the mechanistic basis of *Chuanxiong Rhizoma*, providing a rationale for future experimental and clinical investigation.

## 2. Materials and methods

### 2.1. Overall study design

In the course of this study, a comprehensive bioinformatics strategy was employed to identify potential key target genes and potential bioactive compounds of *Chuanxiong Rhizoma* relevant to the treatment of VaD. Target genes associated with *Chuanxiong Rhizoma* were obtained through integration of multiple TCM databases, including the Traditional Chinese Medicine Systems Pharmacology Database and Analysis Platform (TCMSP), the Encyclopedia of Traditional Chinese Medicine (ETCM), SymMap, BATMAN-TCM, HERB, and the Herbal Ingredients’ Targets Platform (HIT) 2.0. VaD-related genes were curated from gene-centric databases such as DisGeNET, GeneCards, and Online Mendelian Inheritance in Man (OMIM). To identify critical target genes, WGCNA, differential expression analysis, single-cell RNA sequencing analysis, and ROC curve analysis were applied to publicly available RNA expression datasets. A GSEA was conducted to investigate the biological functions and pathway connections of the identified potential key targets. Additionally, immune cell infiltration analysis was conducted using mRNA expression profiles from VaD cohorts to assess the correlation between phenotype-associated target genes and immune cell abundance. Network pharmacology was utilized to predict the interactions of *Chuanxiong Rhizoma*’s active constituents with the core target genes related to the pathogenesis of VaD. Predicted compound-target interactions were further evaluated via molecular docking and dynamics simulations to assess the stability of binding. The overall workflow is summarized in Figure 1.

**Figure 1.**
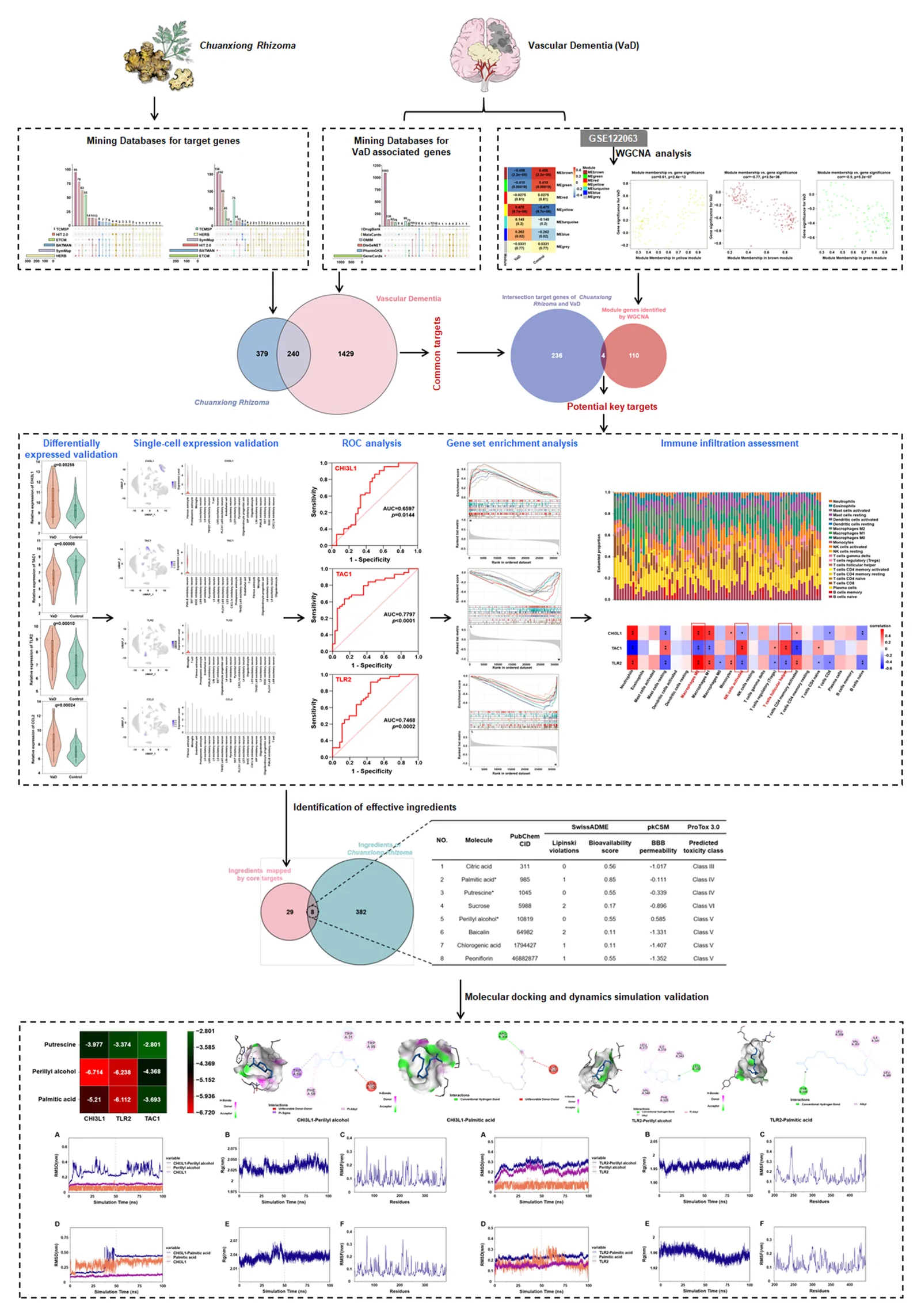
A flowchart illustrating the research workflow for identifying potential active ingredients from *Chuanxiong Rhizoma* in the treatment of vascular dementia (VaD), with CHI3L1 and TLR2 highlighted as potential key therapeutic targets. VaD = vascular dementia.

### 2.2. Screening of potential active constituents and corresponding target genes of Chuanxiong Rhizoma

To identify the bioactive compounds and their corresponding target genes associated with *Chuanxiong Rhizoma*, this study integrated data from 6 publicly available pharmacological databases based on predefined screening criteria and parameter thresholds. These databases included the SymMap database,^[20]^ the BATMAN-TCM online bioinformatics analysis tool (BATMAN),^[21]^ TCMSP,^[22]^ ETCM,^[23]^ the HERB database,^[24]^ and HIT 2.0.^[25]^ After merging the results, duplicate target genes were removed, and nonredundant targets were maintained for further examination.

### 2.3. Screening of VaD-associated genes

To identify potential target genes associated with VaD, this study systematically collected data from 6 authoritative disease-gene databases using the keyword “VaD.” These databases included DrugBank (https://go.drugbank.com/), MalaCards,^[26]^ OMIM (https://www.omim.org/), DisGeNET,^[27]^ Pharmacogenetics and Pharmacogenomics Knowledge Base (PharmGKB) (https://www.pharmgkb.org/), and GeneCards (https://www.genecards.org/). To improve the specificity and relevance of the identified targets, predefined filtering criteria were applied: only genes with a relevance score >10 from MalaCards and GeneCards and a score >0.05 from DisGeNET were retained. All entries from OMIM, DrugBank, and PharmGKB were included without additional filtering due to their high curation standards. After merging the results from all databases, duplicate genes were removed, and targets without redundancy were preserved for subsequent analysis.

### 2.4. Acquisition of intersecting target genes related to Chuanxiong Rhizoma and VaD

To explore the shared molecular targets between VaD and *Chuanxiong Rhizoma*, an intersection analysis was conducted between the 2 sets of target genes using an online Venn diagram tool (https://www.bic.ac.cn/test/venn/#/), which enables comparative visualization of overlapping elements. The resulting common target genes were identified and retained for subsequent downstream analysis.

### 2.5. Screening of significant module genes by WGCNA

The public dataset GSE122063, comprising brain tissue samples from 44 healthy controls and 36 individuals diagnosed with VaD, along with 56 patients with AD, was retrieved from the Gene Expression Omnibus (GEO) database (https://www.ncbi.nlm.nih.gov/geo/query/acc.cgi?acc=GSE122063). The study examined gene expression data from 36 patients with VaD and 44 healthy individuals. WGCNA was utilized to establish signed co-expression networks based on scale-free topology criteria using the biweight midcorrelation (Bicor) algorithm. For network construction, the top 2000 genes with the highest mean absolute deviation were selected, and outlier samples were removed with a detection threshold set to −2.5. To identify the optimal soft threshold power for a scale-free network, the pickSoftThreshold function from the WGCNA R package was utilized to evaluate powers from 1 to 30. The smallest power at which the scale-free topology fit index reached 0.85 was selected. A random seed of 2020 was set for reproducibility. Subsequently, transformation of the adjacency matrix into a topological overlap matrix was performed and hierarchical clustering was utilized with the corresponding dissimilarity measure to generate a cluster dendrogram. Module detection was carried out using a dynamic tree-cutting algorithm with a minimum module size requirement of 25 genes, a deep split of 2, and an edge weight threshold of 0.5. Highly similar modules were merged using a merge cut height of 0.2. Modules significantly linked to VaD were identified based on module-trait relationships, and genes within these modules were screened by calculating (|module membership| > 0.8) and (|gene significance| > 0.2). Genes satisfying both thresholds were defined as significant module genes. These significant module genes were then intersected with the previously identified shared target genes related to *Chuanxiong Rhizoma* and VaD to obtain candidate therapeutic target genes (Table S1, Supplemental Digital Content 1).

### 2.6. Differential expression analysis of candidate target genes

The candidate target genes were validated for differential expression using microarray data from the frontal and temporal cortex samples in dataset GSE122063, which included 44 control samples and 36 VaD samples. This analysis was restricted to the 4 prespecified candidate genes, no genome-wide differential expression testing was performed. Gene expression levels were summarized as mean ± standard deviation. Differential expression analysis was conducted by an independent samples *t* test in SPSS version 26, with Levene’s test used to assess homogeneity of variance and Welch’s correction applied when variances were unequal. To account for multiple comparisons across the 4 candidate genes, the Benjamini-Hochberg (BH) procedure was applied, and a BH-adjusted *q*-value below 0.05 was deemed statistically significant. Violin plots were used to visualize the results to illustrate expression distribution across groups. Candidate genes showing significant differential expression after BH correction were selected for subsequent functional and mechanistic validation.

### 2.7. Single-cell expression analysis of candidate target genes

The Deeply Integrated human Single-Cell Omics (DISCO) platform (available at https://www.immunesinglecell.org/) is a comprehensive resource designed to facilitate the exploration of published single-cell datasets and enable integrated analysis with user-submitted data. It provides high-resolution reference maps for assessing distinct gene expression profiles for each cell type at the single-cell level. In this study, DISCO was used to verify the expression profiles of candidate target genes in human brain tissues, allowing evaluation of their cellular distribution and potential functional relevance within specific neural and glial cell populations. Conversely, candidate genes showing no detectable expression or clear cell type enrichment across the analyzed human brain cell populations were considered unsupported at the single-cell level and were therefore not carried forward into the subsequent compound-target analyses.

### 2.8. ROC analysis of crucial target genes

ROC analysis was employed to assess the capability of candidate target genes to distinguish VaD from healthy controls based on sensitivity and specificity. The diagnostic potential of each gene was evaluated through calculation of the area under the curve (AUC), with an AUC exceeding 0.6 indicating predictive power above random expectation. Using gene expression data from GSE122063, AUC values were computed for each candidate gene to quantify its suitability as a diagnostic biomarker. The statistical significance of each AUC was determined using the Wilson score method, and differences in levels of expression between the VaD and control groups were compared to validate the biological relevance of identified genes. Nevertheless, a statistically significant AUC was not used as the sole criterion for retaining a candidate target; genes lacking predicted interactions with *Chuanxiong Rhizoma* constituents, or showing unfavorable docking affinities toward all tested compounds, were de-prioritized in the subsequent compound-target analyses.

### 2.9. Gene set enrichment analysis of crucial target genes

The GSE122063 cohort was analyzed with GSEA software (version 4.1.0) focusing on the expression profiles of candidate target genes. GSEA was performed using KEGG gene sets. The top 10 enriched gene sets were identified according to the following criteria: a normalized enrichment score <–1 or higher than 1, a nominal *P*-value below .05, and a false discovery rate <0.25. The pathways that are significantly enriched highlight biological processes and molecular functions connected to the expression of candidate target genes in VaD.

### 2.10. Immune infiltration assessment of crucial target genes

The Cell-type Identification by Estimating Relative Subsets of RNA Transcripts (CIBERSORT), a computational deconvolution tool based on bulk‑tissue gene‑expression profiles, was used to estimate the relative proportions of immune‑cell subsets in VaD patients and healthy controls.^[28]^ Samples with a CIBERSORT deconvolution *P*-value ≥.05 were excluded prior to group comparison. CIBERSORT was run using the LM22 signature matrix with 1000 permutations. This deconvolution algorithm enables the estimation of the relative abundance of immune cell subsets from heterogeneous tissue samples. Using the CIBERSORT implementation in R, the relative fractions of the 22 immune cell subsets were estimated for both the VaD and control cohorts. The Wilcoxon rank-sum test was utilized to assess variations in immune cell proportions across groups, with a significance threshold of *P* < .05. Box plots were employed to visualize the distribution of immune cell compositions across different immunological profiles. The expression of candidate target genes and their association with specific immune cell types were evaluated through Spearman correlation analysis to assess potential immunomodulatory roles in VaD.

### 2.11. Identification of effective compounds interacting with crucial targets

Four pharmacologically relevant databases, including HIT 2.0, BATMAN-TCM, TCMSP, and HERB, were integrated to identify effective compounds of *Chuanxiong Rhizoma* that potentially target the crucial genes associated with VaD. Duplicate compounds were removed, and nonredundant entries were retained. To determine the overlapping compounds shared between *Chuanxiong Rhizoma* and the crucial target genes, a Venn diagram was constructed using an online visualization tool (https://www.bic.ac.cn/test/venn/#/). Subsequently, a set of drug-likeness and pharmacokinetic criteria was applied for compound filtering: Lipinski violations ≤1, oral bioavailability score ≥0.1, blood–brain barrier (BBB) permeability ≥−1, and predicted toxicity class ≥IV. Compounds meeting all these criteria were preserved for downstream analysis.

### 2.12. Molecular docking analysis

Molecular docking calculations were performed using AutoDock Vina to determine the binding affinity of bioactive ingredients from *Chuanxiong Rhizoma* with their corresponding target proteins. Although no compound‑target annotation for CHI3L1 was available from the input databases, we included CHI3L1 for docking analysis given its shared inflammatory signaling axes with TLR2. Two-dimensional chemical structures of ingredients were collected from the PubChem database (https://pubchem.ncbi.nlm.nih.gov/). The 3-dimensional structures of CHI3L1 (PDB ID: 8R4X) and TLR2 (PDB ID: 2Z7X) were obtained from the RCSB Protein Data Bank (PDB), and the structure of TAC1 (AlphaFold model AF-P20366-F1) was obtained from the AlphaFold Protein Structure Database (https://alphafold.ebi.ac.uk/). To prepare protein structures for docking, water molecules and heteroatoms were eliminated, polar hydrogen atoms were introduced, and Gasteiger charges were assigned using PyMOL (https://pymol.org/2/) and AutoDock Tools. All processed structures were altered to PDBQT format for subsequent molecular docking simulations. Docking calculations were conducted using AutoDock Vina with default exhaustiveness (8) and num_modes set to 10. Because no experimentally characterized ligand-binding pocket was available for CHI3L1, TAC1 or TLR2, docking was performed as a whole-protein blind search: for each receptor, the grid box was centered on the protein and sized to enclose it. The lowest-energy binding pose was selected for interaction analysis. The binding poses obtained were displayed using PyMOL and Discovery Studio 2020. Both 2-dimensional interaction diagrams and 3-dimensional structural models were generated to illustrate the molecular interactions. The strength of interactions between compounds and targets was evaluated using binding affinity values. Binding affinity thresholds were defined as follows: <−7.0 kcal/mol, strong binding; −5.0 to −7.0 kcal/mol, moderate binding; >−5.0 kcal/mol, weak binding, with more negative values indicating stronger binding. These thresholds are commonly used in molecular docking studies employing the AutoDock Vina scoring function.^[2]^

### 2.13. Molecular dynamics simulation analysis

The protein-ligand complex, resulting from molecular docking studies, was subjected to molecular dynamics simulations using GROningen MAchine for Chemical Simulations 2022. The small-molecule ligand underwent parameterization via the General Amber Force Field, while the protein was described by the AMBER14SB force field. The system was solvated with water molecules modeled by the transferable interatomic potential with three points model water model. The protein and ligand files were combined to build the simulation system, which was subsequently neutralized by adding counterions and subjected to energy minimization. Simulations were conducted under constant temperature (298 K) and pressure (1 bar) conditions using periodic boundary conditions. The LINCS algorithm was employed to constrain covalent bonds with hydrogen atoms, employing a 2 femtosecond integration time step. The Particle Mesh Ewald method was performed to calculate electrostatic interactions with a cutoff of 1.2 nm, whereas nonbonded interactions were truncated at 10 Å and updated every 10 steps. The V-rescale thermostat ensured temperature stability, and the Berendsen barostat regulated pressure coupling during the equilibration phase. Prior to the production simulation, the system was stabilized by conducting 100 ps of NVT and 100 ps of NPT equilibration runs. This was followed by a 100 nanosecond (ns) production molecular dynamics simulation, with trajectories saved every 10 ps for analysis. Post-simulation analysis was performed using VMD and PyMOL. Binding stability was evaluated through the calculation of the root mean square deviation (RMSD) for the protein backbone and ligand, radius of gyration (Rg) of the protein, and root mean square fluctuation (RMSF) per residue.

## 3. Results

### 3.1. Analysis of intersection target genes between Chuanxiong Rhizoma and VaD

To identify the common target genes between *Chuanxiong Rhizoma* and VaD, UpSet plots were employed in this study. As illustrated in Figure 2A–B, these UpSet plots visualize the distribution of 390 bioactive compounds and their 619 corresponding target genes derived from TCMSP, HIT2.0, ETCM, BATMAN, SymMap, and HERB databases. Figure 2C presents the UpSet plot depicting the distribution of 1669 genes linked to VaD retrieved from the DrugBank, MalaCards, OMIM, DisGeNET, PharmGKB, and GeneCards databases. Subsequently, a Venn diagram analysis revealed 240 overlapping target genes between *Chuanxiong Rhizoma* and VaD as illustrated in Figure 2D (Table S2, Supplemental Digital Content 2). These shared targets indicate potential molecular mechanisms through which *Chuanxiong Rhizoma* may exert therapeutic effects against VaD.

**Figure 2.**
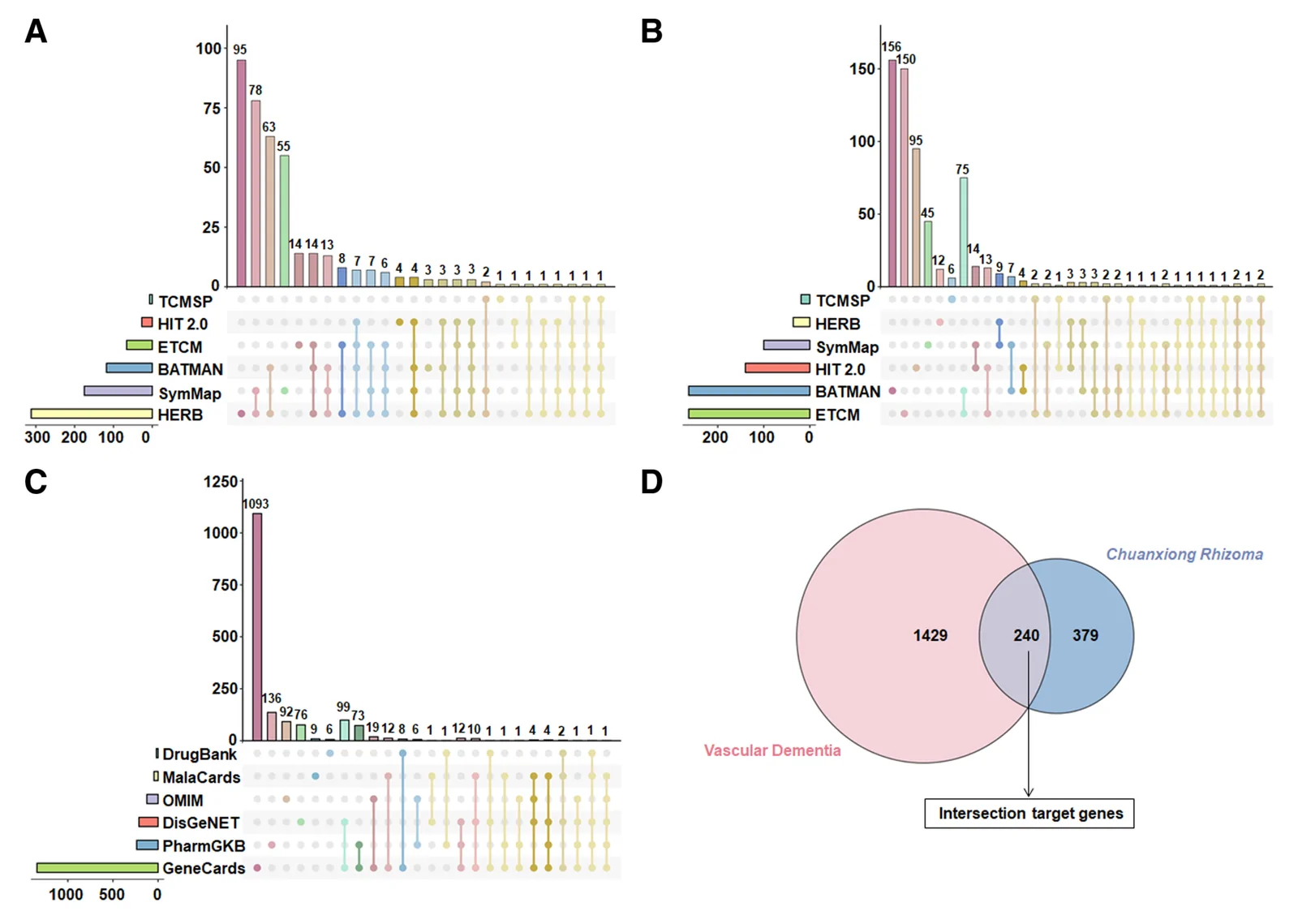
Analysis of intersecting target genes between *Chuanxiong Rhizoma* and vascular dementia (VaD). (A) An UpSet plot showing the bioactive compounds identified in *Chuanxiong Rhizoma*. (B) An UpSet plot displaying the target genes associated with *Chuanxiong Rhizoma*. (C) An UpSet plot presenting the disease-associated genes linked to VaD. (D) A Venn diagram illustrating the overlapping target genes shared by *Chuanxiong Rhizoma* and VaD. In panels A to C, the horizontal bar on the left represents the total number of compounds or target genes identified for each dataset. Dots and connecting lines indicate specific subsets of compounds or genes, while the vertical histogram quantifies the number of elements within each subset. VaD = vascular dementia, TCM = traditional Chinese medicine, HIT = the Herbal ingredients’ targets platform.

### 3.2. Identification of significant module genes using WGCNA

Gene expression profiles from VaD and control groups in a frontal and temporal cortex microarray dataset (GSE122063) were used to build co-expression networks via the WGCNA package. A scale-free network was created by choosing a soft-thresholding power (β = 9) based on the scale-free topology criterion (*R*^2^ = 0.85) (Fig. 3A). Dynamic tree-cutting algorithms paired with hierarchical clustering were applied to identify distinct gene modules (Fig. 3B). Merging highly similar modules yielded 7 functionally distinct gene clusters, which are presented as a dendrogram (Fig. 3C). Among these, the yellow, brown, and green modules showed significant associations with VaD status. Specifically, the yellow module displayed a positive correlation (correlation coefficient = 0.61, *P* = 2.4e–12), while the brown (correlation coefficient = −0.77, *P* = 3.5e–36) and green (correlation coefficient = −0.5, *P* = 5.2e–07) modules displayed strong negative correlations with VaD (Fig. 3D). Genes from these 3 significantly associated modules were integrated to derive the WGCNA results, leading to the identification of 114 potential key module genes (Figure 4A, left).

**Figure 3.**
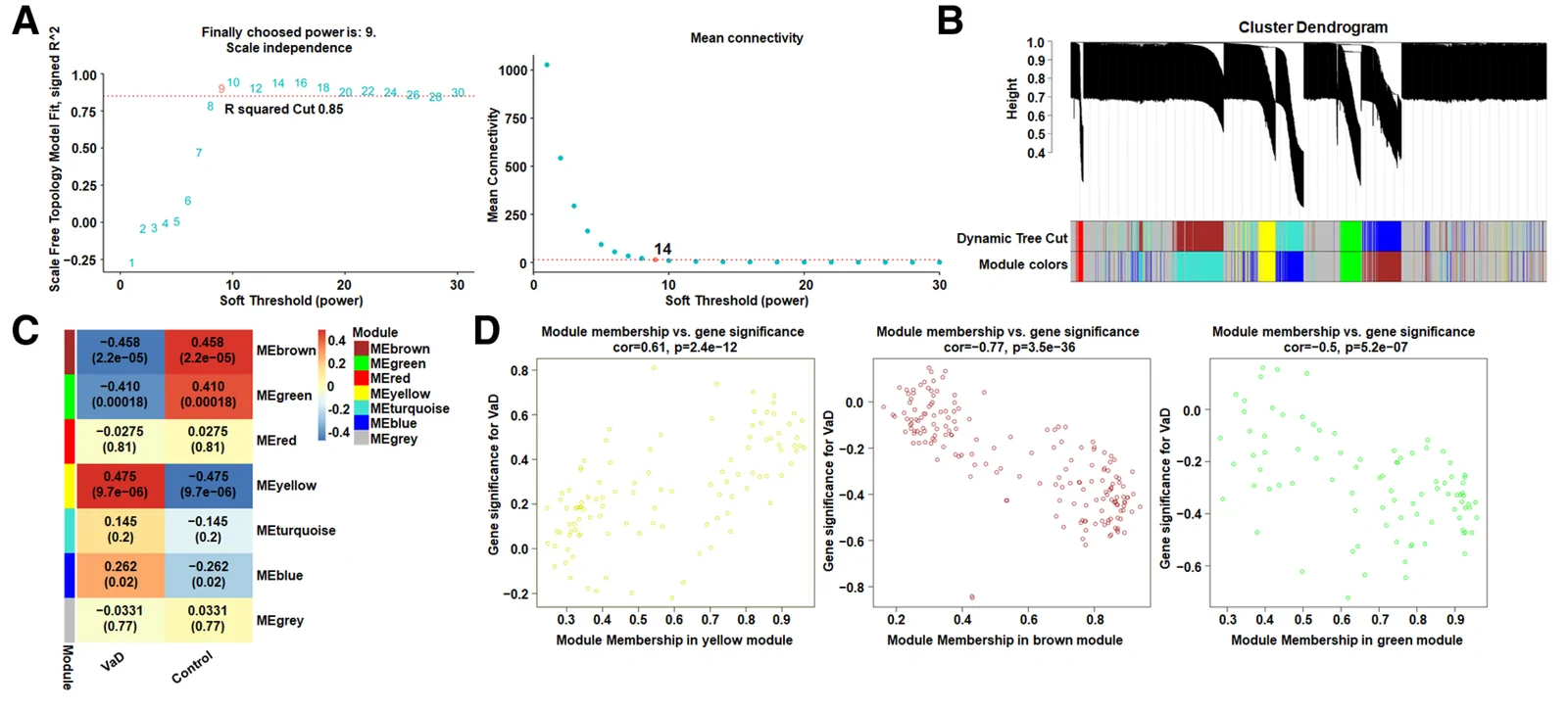
Identification of significant module genes through weighted gene co-expression network analysis (WGCNA). (A) Plot showing the soft threshold power (left) and scale-free topology fit (right) used to determine the optimal power for network construction. (B) Hierarchical clustering dendrogram of all genes based on topological overlap, representing the gene co-expression network structure. (C) Heatmap displaying the correlation between module genes and clinical traits, vascular dementia versus normal controls, with corresponding *P*-values indicated. (D) Scatter plots illustrating the relationship between gene significance (GS) and module membership (MM) in the yellow, brown, and green modules, where higher MM values indicate stronger intramodular connectivity. VaD = vascular dementia.

**Figure 4.**
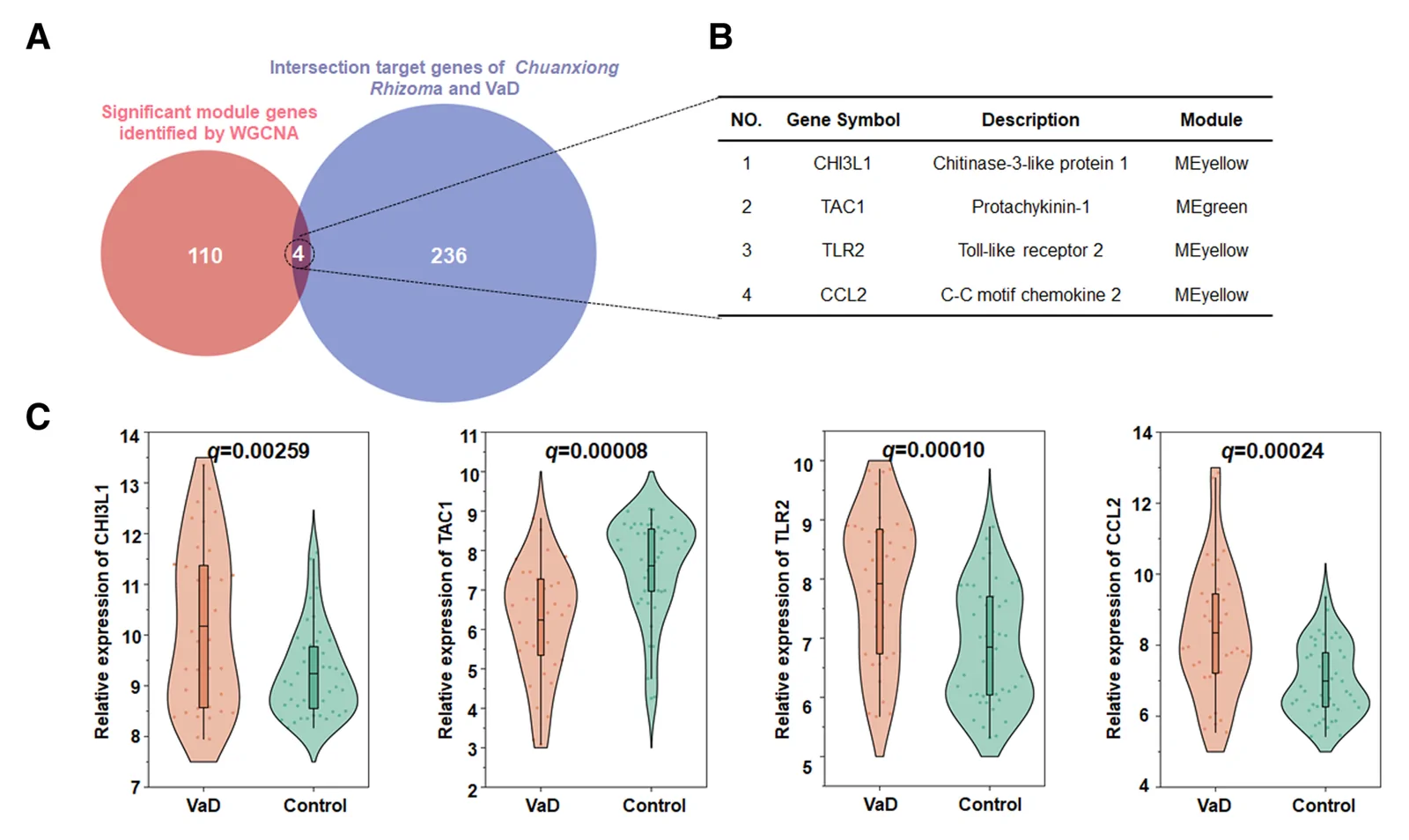
Identification of candidate target genes. (A) Venn diagram illustrating the 2 complementary strategies used to identify candidate target genes: intersection of *Chuanxiong Rhizoma* targets and VaD-associated genes, and module-trait associations from WGCNA. (B) Detailed annotation of 4 candidate target genes, including gene symbols, description, and modules. (C) Comparative expression analysis of the candidate target genes between control and VaD groups in the GSE122063 dataset. The control group (N = 44) and VaD group (N = 36) are shown. Data are presented as violin plots with means ± standard deviation; statistical significance was assessed using the independent samples *t* test, with BH-adjusted *q* < 0.05 considered significant. VaD = vascular dementia, WGCNA = weighted gene co-expression network analysis.

### 3.3. Identification of candidate target genes and validation using the GEO dataset

To further identify candidate target genes, Venn diagrams were utilized to intersect the shared targets between *Chuanxiong Rhizoma* and VaD with the significant module genes derived from WGCNA, yielding 4 overlapping genes: CHI3L1, TAC1, TLR2, and CCL2 (Figs. 4A–B). In contrast with the control group, the expression amounts of these 4 genes showed distinct and statistically significant alterations in VaD patients. Specifically, CHI3L1, TLR2, and CCL2 were significantly increased in the VaD group, while TAC1 exhibited a downward trend (Fig. 4C). These results indicate that CHI3L1, TLR2, TAC1, and CCL2 may serve as potential therapeutic targets through which *Chuanxiong Rhizoma* may exert potential effects in VaD.

### 3.4. Single-cell annotation and expression validation of candidate target genes in different brain cells

To ascertain the primary cellular sources of the candidate target genes in brain tissue, single-cell analyses were performed using the DISCO database. As illustrated in Figure 5, CHI3L1 was predominantly expressed in fibrous astrocytes, TAC1 was mainly localized in Parvalbumin inhibitory neurons, and TLR2 was primarily detected in microglia, indicating cell type-specific expression patterns. In contrast, CCL2 showed no detectable expression in any of the analyzed brain cell types and was therefore excluded from the subsequent compound-target analyses. These findings suggest that CHI3L1, TAC1, and TLR2, which were identified as potential targets, are selectively expressed in key brain cell populations implicated in VaD pathogenesis, supporting their roles in the therapeutic mechanism of *Chuanxiong Rhizoma*.

**Figure 5.**
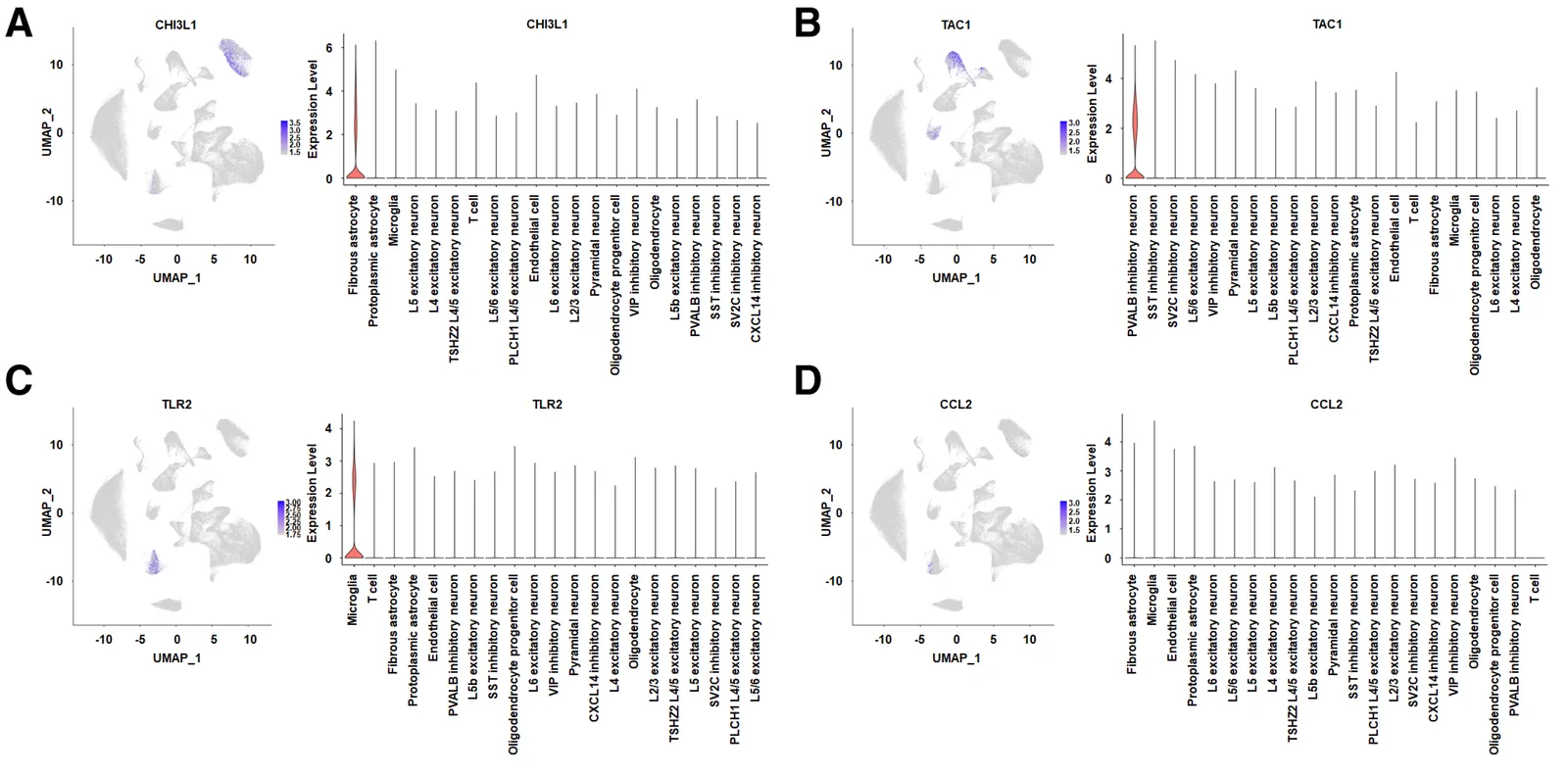
Single-cell annotation and expression analysis of candidate target genes across different brain cell types. (A) CHI3L1 expression is predominantly localized in fibrous astrocytes. (B) TAC1 expression is primarily enriched in PVALB inhibitory neurons. (C) TLR2 expression is mainly detected in microglia. (D) CCL2 expression does not show specific enrichment in any particular brain cell type. UMAP = Uniform Manifold Approximation and Projection.

### 3.5. ROC curve analysis of CHI3L1, TAC1, and TLR2

ROC curve analysis was performed using a gene expression dataset from the frontal and temporal cortex (GSE122063) to evaluate the diagnostic properties of the selected target genes, CHI3L1, TAC1, and TLR2, in VaD, as shown in Figure 6A. The AUC is used to assess diagnostic accuracy, with values close to 1 signifying excellent performance. Our results indicated that CHI3L1 (AUC = 0.6597, *P* = .0144), TAC1 (AUC = 0.7797, *P* < .0001), and TLR2 (AUC = 0.7468, *P* = .0002) exhibit statistically significant AUC values, reflecting considerable sensitivity and specificity for VaD diagnosis. Moreover, as illustrated in Figure 6B, these results are consistent with earlier research implicating CHI3L1, TAC1, and TLR2 in VaD pathogenesis. These results support the hypothesis that CHI3L1, TAC1, and TLR2 may serve as key mediators of the therapeutic effects of *Chuanxiong Rhizoma* in VaD pending validation in independent cohorts.

**Figure 6.**
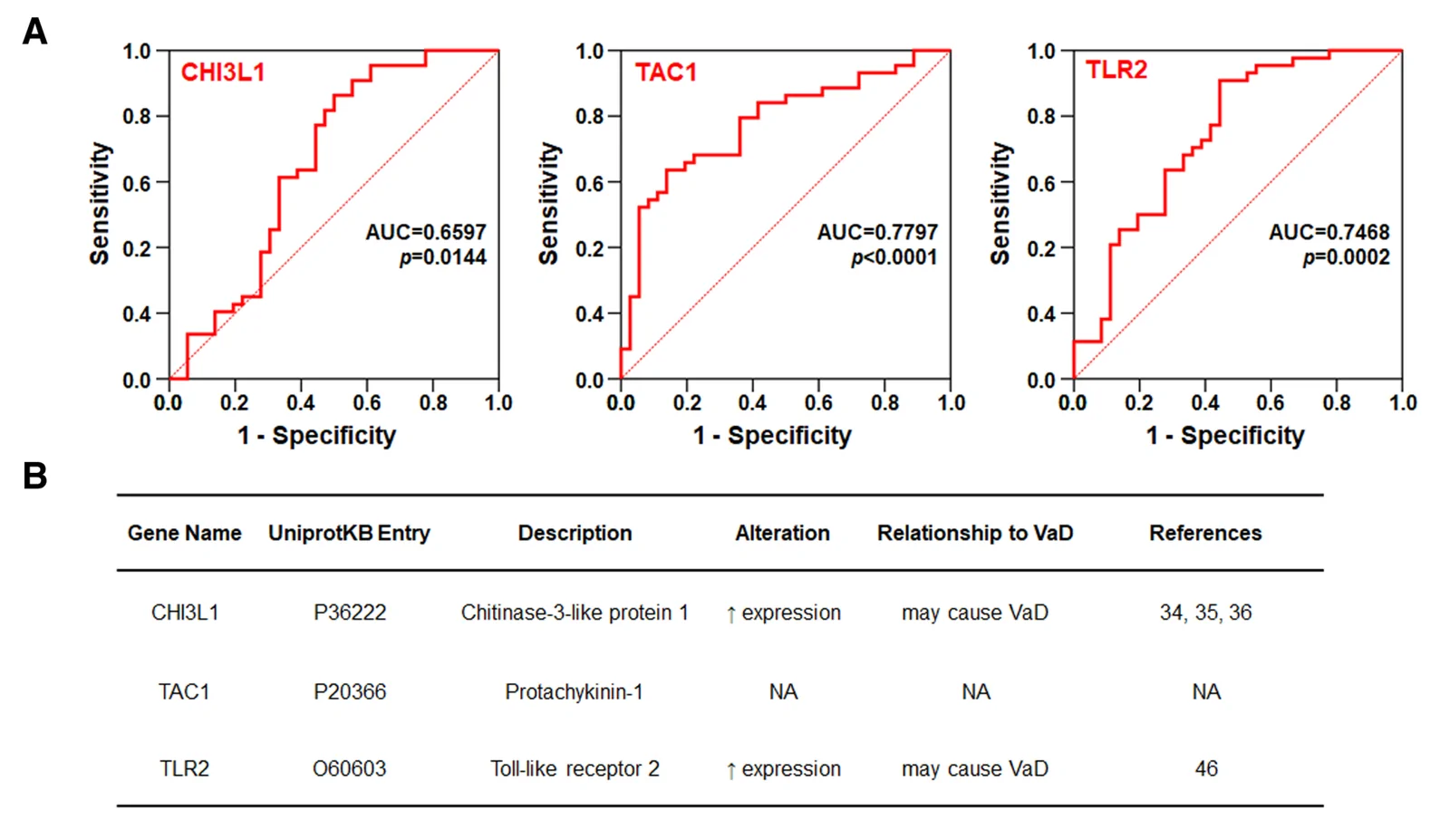
Receiver operating characteristic (ROC) analyses of potential key target genes. (A) ROC curves demonstrating the clinical predictive performance of candidate target genes based on the GEO dataset GSE122063 from the human frontal and temporal cortex. The analysis included samples from a control group (N = 44) and a vascular dementia (VaD) group (N = 36), with statistical significance set at *P* < .05. The area under the curve (AUC) value was used to evaluate diagnostic accuracy. (B) Summary of critical target genes associated with VaD, including gene name, description, and annotations. AUC = area under the curve, GEO = the gene expression omnibus, ROC = receiver operating characteristic, VaD = vascular dementia.

### 3.6. GSEA functional analyses of target genes

To gain a clearer insight into the roles of selected target genes in VaD, GSEA was used to investigate the biological pathways linked to CHI3L1, TAC1, and TLR2. CHI3L1 was notably involved in pathways such as Fc gamma R-mediated phagocytosis, B Cell receptor signaling, and apoptosis (Fig. 7A). TLR2 was predominantly involved in Fc gamma R-mediated phagocytosis, B Cell receptor signaling, leishmania infection, and others (Fig. 7B). TAC1 showed significant enrichment in pathways associated with Parkinson’s disease, Huntington’s disease, oxidative phosphorylation, and others (Fig. 7C). Notably, CHI3L1, TAC1, and TLR2 were commonly enriched in the chemokine signaling pathway, allograft rejection, and the JAK/STAT signaling pathway (Fig. 7D). These shared inflammatory and immune-related pathways suggest that modulation of neuroinflammation may contribute to the remedial effects of *Chuanxiong Rhizoma* in VaD.

**Figure 7.**
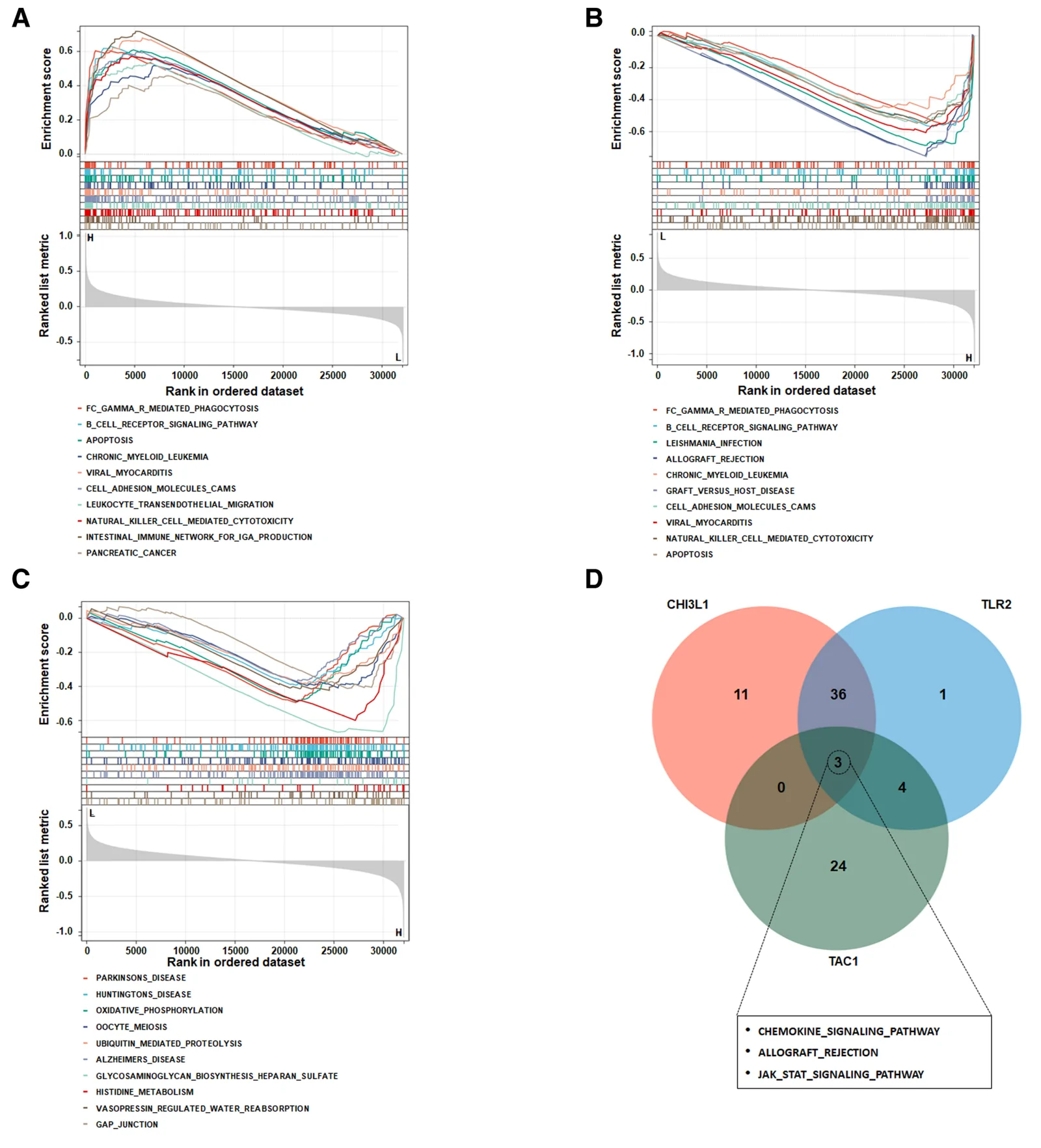
Gene set enrichment analysis of potential key target genes. (A) CHI3L1, (B) TLR2, and (C) TAC1. (D) Venn diagram illustrating the shared pathways among the gene sets.

### 3.7. Analysis of immune cell infiltration

Given the immune‑related pathways uncovered in the above findings, we next examined whether the immune cell composition estimated from bulk brain transcriptomes differed between VaD and control samples. To explore alterations in the immune landscape associated with VaD, immune cell infiltration was analyzed using CIBERSORT to evaluate the relative percentages of 22 specific immune cell types within the collected samples (Fig. 8A). LM22 was defined from peripheral immune cells and does not contain a microglial signature; the fractions reported here are therefore relative estimates from bulk tissue, not direct measurements of central nervous system (CNS)–resident or infiltrating cells. Notably, patients with VaD exhibited significant changes in Macrophages M2, activated natural killer cells (NK cells), and follicular helper T cells compared to controls (Fig. 8B). Furthermore, correlation analyses were performed between each candidate target gene and the infiltrating immune cell populations (Fig. 8C). The correlation of CHI3L1 with Macrophages M2 was significantly positive, whereas it was significantly negative with activated NK cells. The correlation of TAC1 with Macrophages M2 was significantly negative, whereas it was significantly positive with both activated NK cells and follicular helper T cells. The correlation of TLR2 with Macrophages M2 was significantly positive, whereas it was significantly negative with activated NK cells and follicular helper T cells. Taken together, the expression of the candidate genes co-varied with several estimated immune cell fractions in bulk brain tissue, consistent with an immune component in VaD pathobiology.

**Figure 8.**
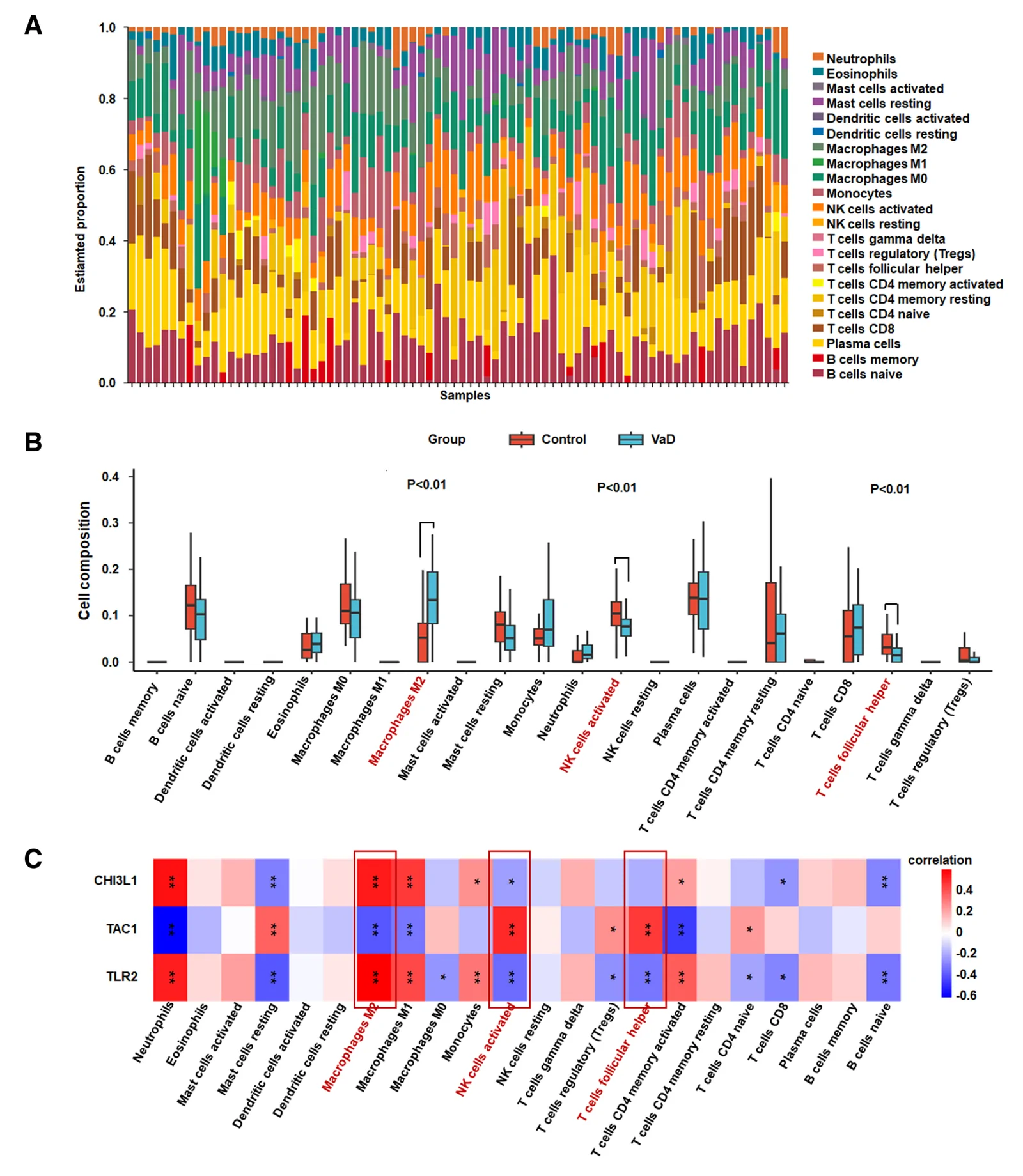
Immune infiltration analyses in the VaD and control groups. (A) Bar plot showing the composition of 22 immune cell types. (B) Histogram depicting differences in immune infiltration levels across the 22 immune cell types between the VaD and control groups. (C) Heatmap displaying correlations between immune infiltrating cells and 3 potential key target genes, with significance levels denoted as * *P* < .05 and ** *P* < .01. VaD = vascular dementia.

### 3.8. Identification of potential bioactive ingredients in Chuanxiong Rhizoma

Which constituents of *Chuanxiong Rhizoma* target the identified genes? To address this question, we integrated data from multiple pharmacological databases, HIT 2.0, BATMAN-TCM, TCMSP, and HERB, to identify potential bioactive compounds targeting CHI3L1, TAC1, and TLR2 (Fig. 9A). To elucidate the connection between active compounds and target genes, an intersection analysis was performed between 390 known components of *Chuanxiong Rhizoma* and 37 gene-related compounds using an online Venn diagram tool (Fig. 9B). This yielded 8 overlapping candidate compounds. These candidates were further filtered through Absorption, Distribution, Metabolism, Excretion, Toxicity prediction platforms, SwissADME, pkCSM, and ProTox 3.0, resulting in 3 high-potential bioactive compounds: palmitic acid, putrescine, and perillyl alcohol (Lipinski violations ≤1, oral bioavailability score ≥0.1, BBB permeability ≥−1, and predicted toxicity class ≥ IV) (Fig. 9C). Notably, no constituent of *Chuanxiong Rhizoma* annotated in these databases was retrieved for CHI3L1 (Fig. 9D). These results highlight the therapeutic possibility of *Chuanxiong Rhizoma* in VaD through specific bioactive components.

**Figure 9.**
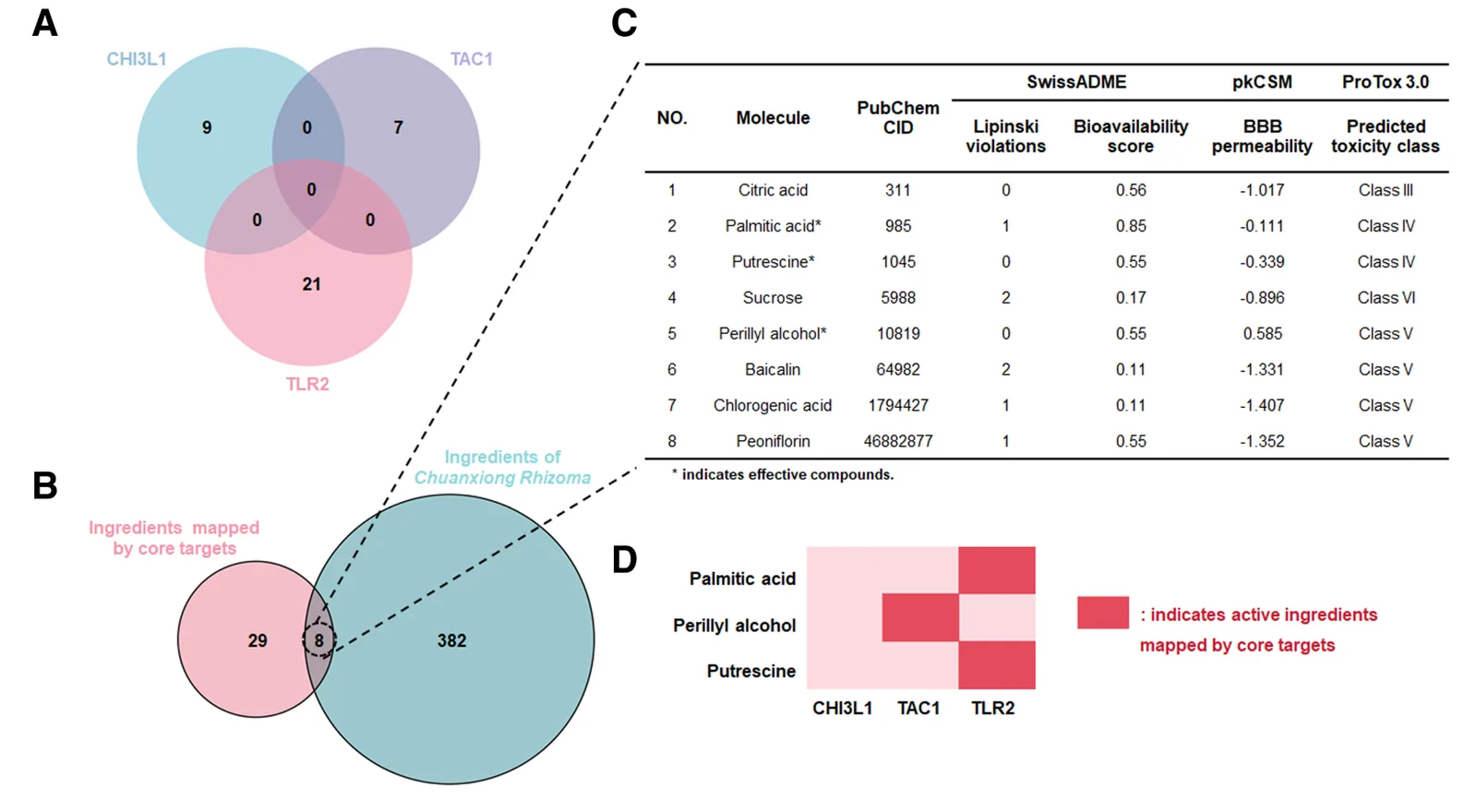
Identification of bioactive ingredients in *Chuanxiong Rhizoma*. (A) Venn diagram showing the number of potential bioactive ingredients associated with 3 potential key targets, as retrieved from the TCMSP, BATMAN-TCM, HERB, and HIT2.0 databases. Light blue represents CHI3L1 (9 ingredients), purple represents TAC1 (7 ingredients), and light red represents TLR2 (21 ingredients). (B) Venn diagram depicting the overlapping ingredients between *Chuanxiong Rhizoma* and the 3 potential key targets. (C) Eight putative active compounds were identified through mapping to the potential key targets, with effective compounds indicated by an asterisk (*). (D) Mapping visualization between the effective ingredients and the potential key target genes. BATMAN = the BATMAN-TCM online bioinformatics analysis tool, TCMSP = the Traditional Chinese Medicine Systems Pharmacology Database and Analysis Platform,

### 3.9. Molecular docking

Molecular docking was performed to explore how the identified bioactive compounds (palmitic acid, perillyl alcohol and putrescine) interact with the candidate target proteins CHI3L1, TAC1, and TLR2 in VaD (Figs. 10A–B). Although no direct ingredient-target prediction was available for CHI3L1 in the databases used, TLR2 and CHI3L1 share inflammatory signaling pathways; we therefore further examined the binding of palmitic acid and perillyl alcohol to CHI3L1. As depicted in Figure 10C, the binding strengths of CHI3L1 were −5.21 kcal/mol for palmitic acid and −6.714 kcal/mol for perillyl alcohol. Similarly, TLR2 showed binding affinities of −6.112 kcal/mol for palmitic acid and −6.238 kcal/mol for perillyl alcohol. In contrast, TAC1 exhibited only limited binding affinity toward the tested compounds (approximately −5 kcal/mol) and was therefore excluded from the subsequent molecular dynamics simulations and compound-target analyses. Additionally, putrescine exhibited poor binding affinity (>−5 kcal/mol) against all 3 targets, CHI3L1, TAC1, and TLR2, indicating weak interaction potential (Fig. 10C). The compound-target interactions were further visualized through PyMOL and Discovery Studio 2020. In the CHI3L1–Perillyl alcohol complex, the binding was stabilized by an unfavorable donor-donor interaction with Asn-100, a Pi–Sigma interaction with Trp-69, and Pi-alkyl interactions with Trp-31, Phe-58, and Trp-99 of CHI3L1 (Fig. 11A). In the CHI3L1-Palmitic acid complex, the interaction involved a conventional hydrogen bond with Arg-304 and an unfavorable donor-donor interaction with Lys-321 (Fig. 11B). For the TLR2-Perillyl alcohol complex, binding was attributed to a conventional hydrogen bond with Leu-350, a Pi-alkyl interaction with Phe-325, and alkyl interactions with Leu-317, Ile-319, Val-343, and Val-348 (Fig. 11C). In the TLR2-Palmitic acid complex, the binding was maintained by a conventional hydrogen bond with Tyr-326 and alkyl interactions with Ile-341, Val-351, Leu-359, and Leu-365 of TLR2 (Fig. 11D). These results indicate that perillyl alcohol and palmitic acid exhibit favorable binding affinities with CHI3L1 and TLR2, respectively, supporting their potential as active constituents. Collectively, these findings suggest that palmitic acid and perillyl alcohol may serve as bioactive components of *Chuanxiong Rhizoma* in the context of VaD, whereas putrescine exhibits limited interaction potential with the target proteins.

**Figure 10.**
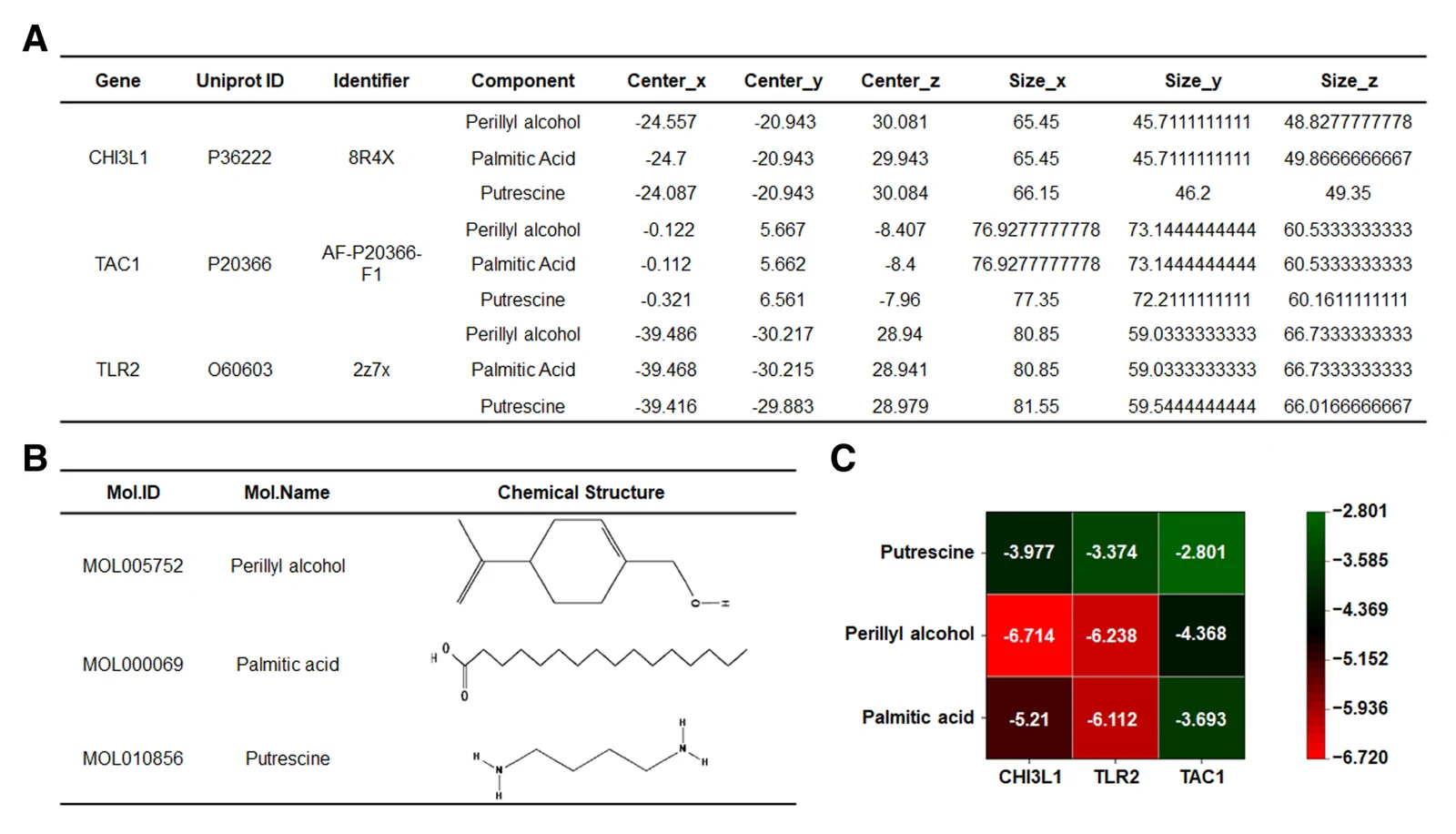
Molecular docking analyses. (A) Candidate target proteins with their UniProt accessions, structure sources and identifiers, together with the grid box center coordinates and dimensions used for each receptor-ligand pair. (B) Effective ingredients of *Chuanxiong Rhizoma* selected for docking analysis. (C) Heatmap illustrating the binding free energy (kcal/mol) derived from molecular docking.

**Figure 11.**
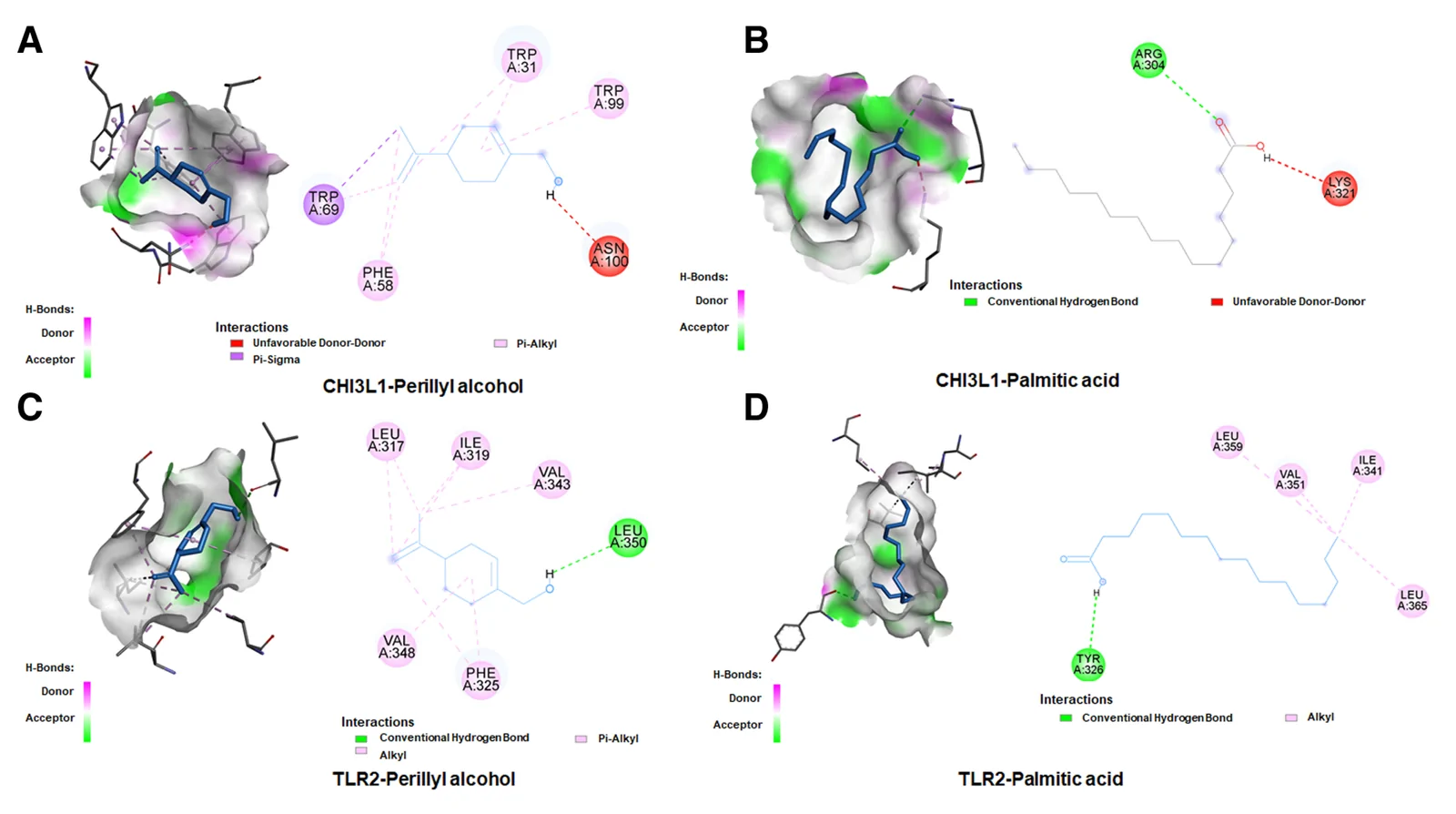
Visualization of docking models. (A) Docking results for the CHI3L1-Perillyl alcohol complex. (B) Docking results for the CHI3L1-Palmitic acid complex. (C) Docking results for the TLR2-Perillyl alcohol complex. (D) Docking results for the TLR2-Palmitic acid complex.

### 3.10. Molecular dynamics simulation

To evaluate the durability of ligand–protein interactions in the CHI3L1-Perillyl alcohol, CHI3L1-Palmitic acid, TLR2-Perillyl alcohol, and TLR2-Palmitic acid complexes, molecular dynamics simulations lasting 100 nanoseconds were performed using GROningen MAchine for Chemical Simulations 2022. RMSD reflects system stability by measuring the average displacement of atoms relative to a reference structure. Rg evaluates structural compactness, where large fluctuations suggest conformational expansion. RMSF quantifies residue-wise flexibility, particularly in side chains. The RMSD profile of the CHI3L1-Perillyl alcohol complex showed pronounced fluctuations throughout the simulation, indicating persistent instability (Fig. 12A). Consistently, the Rg values exhibited significant variability, further supporting structural instability (Fig. 12B). RMSF analysis indicated high flexibility in the side chain regions of several residues (Fig. 12C). In contrast, the RMSD of the CHI3L1-Palmitic acid complex stabilized after approximately 50 ns, suggesting the adoption of a stable conformation (Fig. 12D). Similarly, the Rg values remained relatively constant after 50 ns, confirming structural compactness (Fig. 12E). The RMSF values for this complex also fell within an acceptable range, indicating limited residue flexibility (Fig. 12F). Furthermore, molecular dynamics simulations indicated that TLR2 maintains stable binding with both perillyl alcohol (Figs. 13A–C) and palmitic acid (Figs. 13D–F) over the full simulation period. Overall, 3 complexes, including CHI3L1-palmitic acid, TLR2-perillyl alcohol and TLR2-palmitic acid, adopted and maintained structurally stable conformations throughout the 100-ns simulation, whereas the CHI3L1-perillyl alcohol complex displayed sustained instability and is therefore not considered a supported interaction.

**Figure 12.**
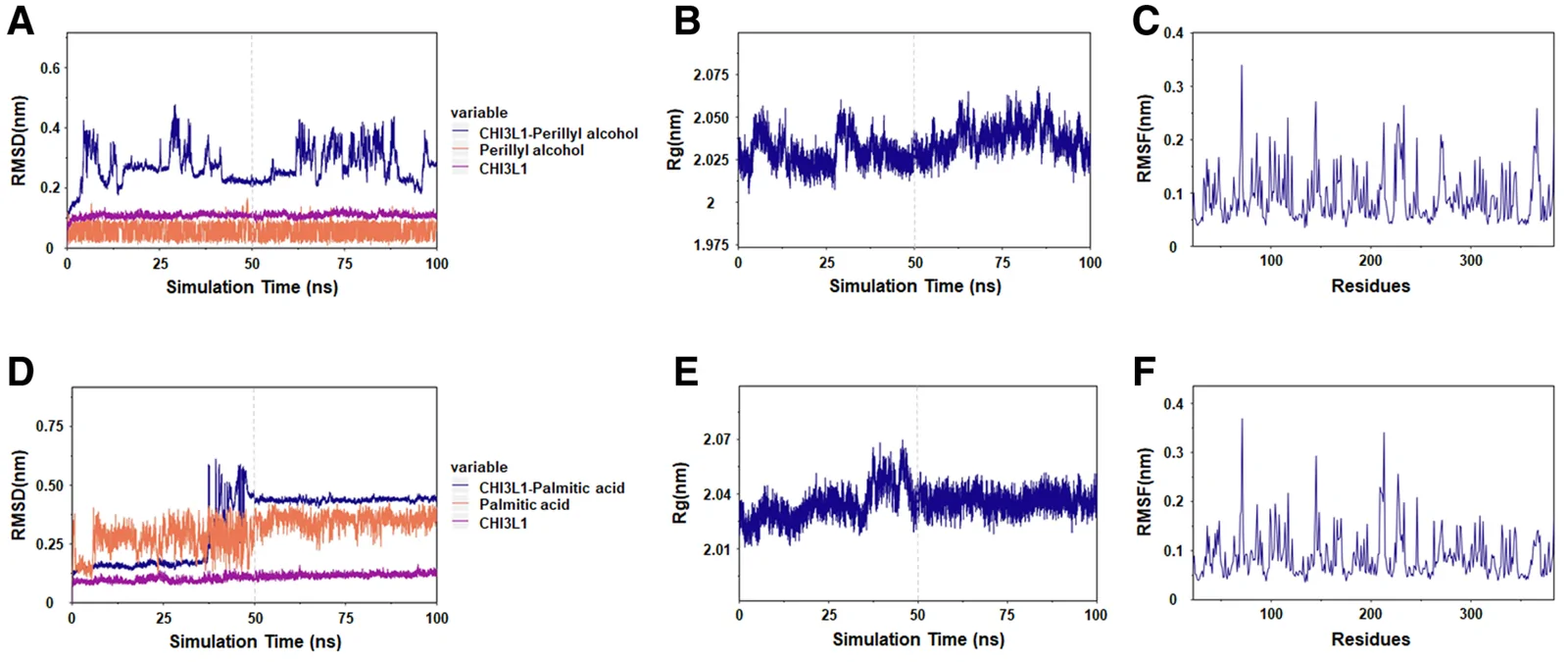
Molecular dynamics simulation analysis of CHI3L1-ligand complexes. (A–C) RMSD, Rg, and RMSF plots for the CHI3L1-Perillyl alcohol complex. The CHI3L1-perillyl alcohol complex is shown for completeness but is not a supported interaction. (D–F) RMSD, Rg, and RMSF plots for the CHI3L1-Palmitic acid complex. RMSD: root mean square deviation, with the x-axis representing time (ns) and the y-axis representing RMSD (nm). Rg: radius of gyration, with the x-axis representing time (ns) and the y-axis representing Rg (nm). RMSF: root mean square fluctuation, with the x-axis representing residue and the y-axis representing RMSF (nm). RMSD = root mean square deviation, RMSF = root mean square fluctuation.

**Figure 13.**
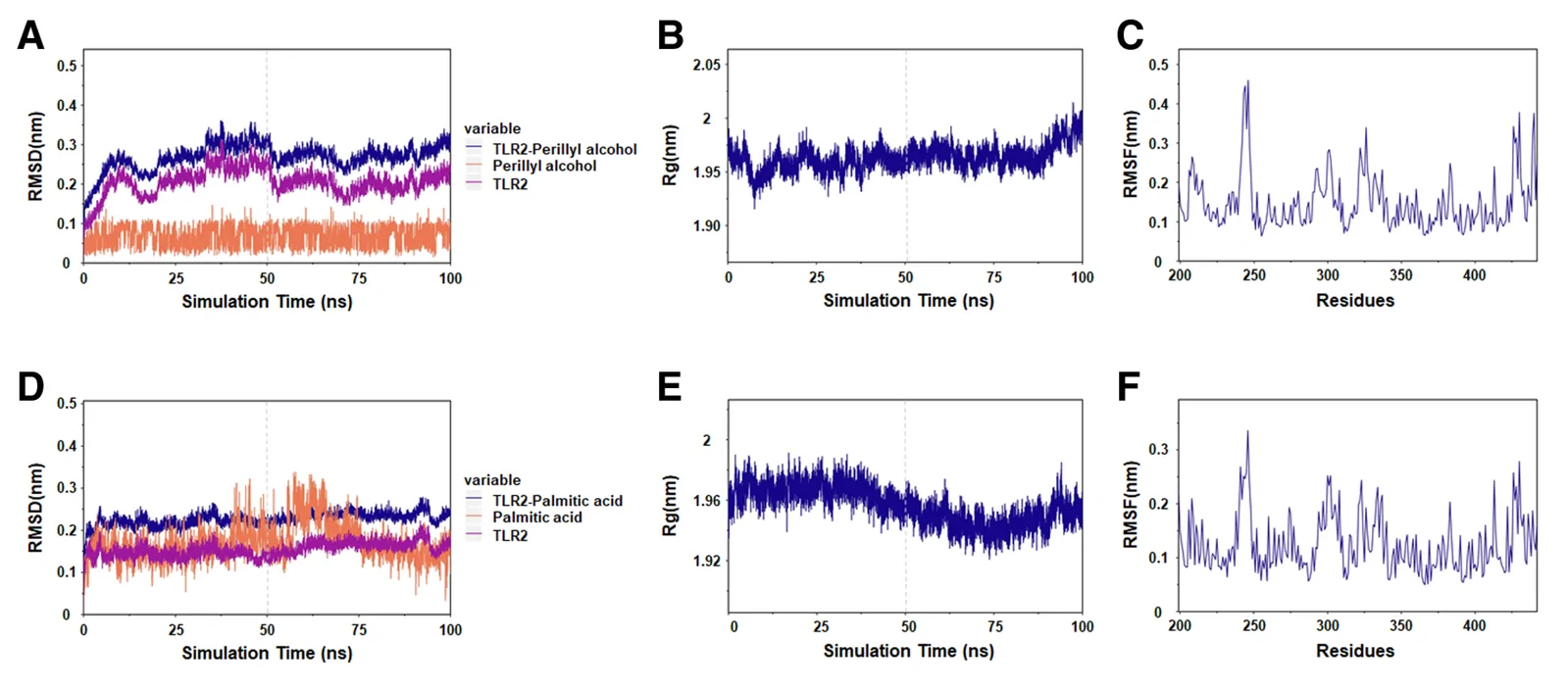
Molecular dynamics simulation analysis of TLR2-ligand complexes. (A–C) RMSD, Rg, and RMSF plots for the TLR2-Perillyl alcohol complex. (D–F) RMSD, Rg, and RMSF plots for the TLR2-Palmitic acid complex. RMSD: root mean square deviation, with the x-axis representing time (ns) and the y-axis representing RMSD (nm). Rg: radius of gyration, with the x-axis representing time (ns) and the y-axis representing Rg (nm). RMSF: root mean square fluctuation, with the x-axis representing residue and the y-axis representing RMSF (nm). RMSD = root mean square deviation, RMSF = root mean square fluctuation.

## 4. Discussion

VaD is a severe condition marked by cognitive impairments resulting from different cerebrovascular disorders and is a significant cause of senile dementia globally. Neuroinflammation in the CNS and blood-brain barrier disruption are established as a key pathogenic factor in VaD, with bidirectional interactions between them. Notably, neuroinflammation can enhance BBB permeability, thereby exacerbating its structural and functional impairment. Conversely, increased BBB permeability can trigger a persistent immune response and sustain a chronic neuroinflammatory state, thus amplifying the pathological cycle of VaD.^[29]^ BBB disruption significantly contributes to neuroinflammation, ultimately leading to neuronal loss. Furthermore, neuroinflammation represents a complex and coordinated response involving all cellular components of the CNS, including microglia, neurons, and infiltrating leukocytes. Chronic cerebral ischemia induces the expansion and activation of glial cells, especially microglia and astrocytes, resulting in an overproduction of cytokines involved in inflammation and reactive oxygen species, both of which are critically implicated in the progression of VaD.^[30]^ Therefore, greater attention should be directed toward the interplay between neuroinflammatory responses and BBB dysfunction in VaD, as targeting these mechanisms may facilitate the development of novel therapeutic agents. By integrating bioinformatics analysis, this study elucidates multi‑target mechanisms of *Chuanxiong Rhizoma* in the treatment of VaD, suggesting that CHI3L1 and TLR2 may serve as potential key targets and that the 2 compounds may bind to these targets, with palmitic acid interacting with both targets and perillyl alcohol with TLR2 in the simulations. Unlike prior network pharmacology studies of VaD, which have analyzed multi-herb formulae and other single herbs and have typically stopped at molecular docking or used molecular dynamics only to corroborate the best-scoring poses,^[31]^ the present work links a data-driven VaD co-expression module to the constituents of a named herb, localizes the candidate genes to defined brain cell types, and applies molecular dynamics as a filter that rejected 1 docking-supported pair. The result is a herb-constrained compound-target hypothesis rather than a biomarker panel.

CHI3L1, alternatively known as YKL-40, is a secreted glycoprotein classified under the glycoside hydrolase family 18, which is a well-established biomarker of immune response and inflammation in peripheral tissues and is predominantly expressed by macrophages, epithelial cells, and endothelial cells.^[32]^ In the brain, CHI3L1 is primarily expressed in astrocytes, particularly in reactive neurotoxic astrocytes induced by microglia.^[32]^ Astrocytes are essential cells in the CNS, responsible for regulating multiple neurophysiological processes. It has been demonstrated that alleviating astrogliosis resulting from vascular stenosis helps maintain white matter integrity and cognitive function.^[33]^ Elevated levels of CHI3L1 are consistently observed in patients with various neurodegeneration diseases, including AD, PD, and VaD.^[34–36]^ Beyond its role as a biomarker, CHI3L1 has been shown to exert direct effects on CNS cells by modulating microglial secretion and activation states, promoting neuronal death, and altering oligodendrocyte precursor cell proliferation.^[37]^ Astrocyte-derived CHI3L1 is linked to the migration of astrocytes, the proliferation of oligodendrocyte progenitor cells, and the differentiation of neural progenitors.^[38]^ Furthermore, CHI3L1 regulates IL-6 expression and induces increases in IL-1β and TNF-α, possibly contributing to BBB disruption and triggering neuroinflammation, eventually causing the loss of neurons.^[39]^ In this study, CHI3L1 expression was considerably higher in the VaD group and predominantly localized in fibrous astrocytes. CHI3L1 exhibited considerable enrichment in pathways such as Fc gamma R-mediated phagocytosis, B Cell receptor signaling, and apoptosis. Moreover, the correlation of CHI3L1 with Macrophages M2 was significantly positive, whereas it was significantly negative with activated NK cells. These findings are consistent with previously reported roles of CHI3L1, supporting its potential as a crucial intervention target for *Chuanxiong Rhizoma* in the treatment of VaD.

Toll-like receptors (TLRs) are mainly found on microglia as well as other immune and nonimmune cells, serving as innate immune receptors that are essential for recognizing external and internal triggers and initiating inflammatory responses.^[40]^ Microglia are innate immune cells essential for physiological processes within the CNS.^[41]^ In patients with VaD, a significant accumulation of phagocytically activated microglia is observed in the perivascular regions surrounding the stroke lesion.^[42]^ Research suggests that TLR2 could contribute to secondary brain damage and ischemia/reperfusion injury by affecting microglial activation and exacerbating neuroinflammation.^[43]^ Upregulation of TLR2 and its signaling pathway is a critical event in focal cerebral ischemia and is associated with increased ischemic damage.^[44]^ Accumulating evidence from recent studies suggests that TLR2 has an important function in disorders related to neurology and neurodegeneration by modulating brain cell function and neural plasticity.^[45]^ TLR2 contributes to VaD pathophysiology by enhancing the pathway of neuronal apoptosis.^[46]^ Therefore, modulation of TLR2 activity may represent a promising therapeutic strategy for effectively managing or even reversing cognitive decline in VaD. In our study, TLR2 expression remarkably increased in the VaD group and predominantly localized in microglia. There was a notable enrichment of TLR2 in pathways including Fc gamma R-mediated phagocytosis, B Cell receptor signaling, leishmania infection, and other immune-related processes. Moreover, the correlation of TLR2 with Macrophages M2 was significantly positive, whereas it was significantly negative with activated NK cells and follicular helper T cells. These findings are consistent with established roles of TLR2, supporting its potential as a crucial intervention target for *Chuanxiong Rhizoma* in the treatment of VaD.

Plant-derived products, including extracts and bioactive compounds, have long been utilized in the treatment of various inflammation-mediated diseases.^[47–49]^ Molecular docking combined with molecular dynamics simulations was employed to identify active ingredients in *Chuanxiong Rhizoma* for the treatment of VaD. Palmitic acid and perillyl alcohol were identified as candidate compounds based on favorable docking scores, acceptable Absorption, Distribution, Metabolism, Excretion, Toxicity profiles and, in 3 of the 4 docked complexes, stable 100-ns MD trajectories. We further note that the CHI3L1-perillyl alcohol complex did not remain stable during MD, and the corresponding claim was not supported. Palmitic acid, a saturated fatty acid, is a structural component of cell membranes and participates in key biological processes such as protein palmitoylation and palmitoylethanolamide synthesis.^[50]^ In Neuro-2a cells, low levels of palmitic acid markedly decrease neuroinflammation by downregulating TNF-α and RhoA GTPase.^[51]^ Previous research has revealed that exposure of N2a cells to palmitic acid markedly reduces lipoteichoic acid-induced TNF-α levels and attenuates TLR2-mediated pro-inflammatory signaling in microglia.^[52]^ Perillyl alcohol, a naturally occurring monoterpene, has been evidenced to possess strong antioxidant, anti-inflammatory, and antiproliferative properties.^[53]^ Treatment with perillyl alcohol suppresses NF-κB nuclear translocation, facilitates the migration of PARKIN into the mitochondria, and preserves cellular redox balance in microglial cells of both mice and humans, thereby reducing the activation of NLRP3 inflammasomes and the processing of caspase-1, IL-1β, and IL-18.^[54]^ An earlier study showed that perillyl alcohol exerts protective effects against the progression from mild cognitive impairment to AD, potentially through inhibition of p-tau181, NF-light, Aβ1-42, Aβ1-40, and GFAP.^[55]^ Further research demonstrated that perillyl alcohol mitigates mitochondrial dysfunction and cytotoxicity caused by Aβ in SH-SY5Y cells, ultimately mitigating neurodegenerative processes associated with AD.^[56]^ Perillyl alcohol has been shown to improve memory formation by affecting dopaminergic neurons and decreasing acetylcholinesterase activity in rats treated with scopolamine, thus alleviating symptoms similar to dementia.^[57]^ These findings suggest that palmitic acid and perillyl alcohol exert therapeutic benefits via multiple targets and pathways, with the core mechanism primarily centered on anti-neuroinflammatory actions. This mechanism is also relevant to the treatment of VaD and aligns with our research findings. Our results support CHI3L1 and TLR2 as potential key therapeutic targets for *Chuanxiong Rhizoma* in the treatment of VaD, highlighting a potential molecular mechanism underlying the neuroprotective effects of this traditional herbal medicine. Furthermore, molecular docking and dynamics simulations illustrated that the CHI3L1-palmitic acid, TLR2-perillyl alcohol, and TLR2-palmitic acid complexes adopt and maintain structurally stable configurations over time, with favorable binding energies and minimal conformational fluctuations. These stable interactions suggest high binding affinity and structural compatibility; however, binding stability alone does not demonstrate functional modulation of either target, and any such activity remains to be established experimentally. These observations suggest that *Chuanxiong Rhizoma* may exert its anti-VaD effects by simultaneously engaging CHI3L1 and TLR2 through 2 bioactive components, palmitic acid and perillyl alcohol, thereby potentially interrupting neuroinflammatory cascades and promoting neural homeostasis. The multi-target nature of this interaction is highly consistent with the holistic pharmacological profile of TCMs, in which the synergistic actions of multiple constituents collectively contribute to therapeutic efficacy. These discoveries not only indicate the traditional use of *Chuanxiong Rhizoma* in the treatment of VaD but also provide a computational rationale for developing future targeted treatments from its bioactive elements.

However, certain limitations of this study should be acknowledged. First, it must be emphasized that the main findings are primarily derived from computational simulations and bioinformatics predictions. Although we have systematically explored the mechanism of action of *Chuanxiong Rhizoma* in VaD through integrated analyses, including WGCNA, differential expression analysis, single-cell RNA sequencing, ROC curve analysis, GSEA, immune infiltration assessment, molecular docking, and molecular dynamics simulations, these results remain largely hypothesis-generating. High docking scores and stable trajectories suggest potential binding interactions but do not confirm actual biological binding activity or functional regulation. The excluded CHI3L1-perillyl alcohol complex illustrated that docking scores alone might produce false-positive interactions. Therefore, the proposed “herb-compound-target-disease” relationships require rigorous validation through experiments conducted in vitro and in vivo to establish their reliability and physiological relevance. Second, the analytical framework relies on public databases, including TCMSP, HIT2.0, ETCM, BATMAN, SymMap, and HERB. A separate constraint concerns the transcriptomic evidence: all expression-based analyses (WGCNA, differential expression, ROC, GSEA, CIBERSORT) rest on GSE122063, the only public human brain bulk VaD transcriptome dataset with matched controls (systematic GEO search performed in September 2026). While these resources are comprehensive, they have inherent limitations regarding data quality, consistency, and completeness. Third, due to modeling feasibility, the network pharmacology approach inevitably involves simplifications of biological system complexity. Our model is static, whereas the biological processes in living organisms are highly dynamic. As such, it cannot accurately simulate the pharmacokinetic behavior of compounds in the body, including absorption, distribution, metabolism, and excretion, nor can it determine whether the predicted bioactive ingredients reach target sites at therapeutically effective concentrations.

## 5. Conclusion

In brief, this study indicated that *Chuanxiong Rhizoma* may exert its anti-VaD effects by simultaneously engaging CHI3L1 and TLR2 through 2 bioactive compounds, palmitic acid and perillyl alcohol. Our study proposes a multi-component, multi-target hypothesis linking candidate bioactive compounds of *Chuanxiong Rhizoma* to CHI3L1 and TLR2 in VaD. Further in vitro and in vivo experimental validation is essential to verify these predicted interactions and to assess any potential therapeutic effects. The findings offer a theoretical basis for the expanded application of *Chuanxiong Rhizoma* in the treatment of VaD and may serve as a reference for guiding future advanced experimental studies.

## Acknowledgments

The data utilized in this research were sourced from publicly accessible databases. A comprehensive list of these sources can be found in the Methods section, and we express our gratitude to the original data contributors.

## Author contributions

**Conceptualization:** Hongchun Liao, Qimin Zeng, Binsheng He.

**Data curation:** Hongchun Liao, Qimin Zeng, Jie Zhang, Taolin Chen, Qi He, Linjie Sun, Jing Wang.

**Methodology:** Hongchun Liao, Qimin Zeng, Juan Li, Qi He.

**Funding acquisition:** Qimin Zeng, Binsheng He, Qin Xiang.

**Supervision:** Binsheng He, Qin Xiang.

**Writing – review & editing:** Binsheng He, Qin Xiang.

**Writing – original draft:** Hongchun Liao, Qimin Zeng.




